# Metal Particle Detection by Integration of a Generative Adversarial Network and Electrical Impedance Tomography (GAN-EIT) for a Wet-Type Gravity Vibration Separator

**DOI:** 10.3390/s23198062

**Published:** 2023-09-24

**Authors:** Kiagus Aufa Ibrahim, Prima Asmara Sejati, Panji Nursetia Darma, Akira Nakane, Masahiro Takei

**Affiliations:** 1 Department of Mechanical Engineering, Division of Fundamental Engineering, Graduate School of Engineering, Chiba University, Chiba 263-8522, Japan; kiagusaufai@gmail.com (K.A.I.); panji.nursetia@chiba-u.jp (P.N.D.); masa2@chiba-u.jp (M.T.); 2 Department of Electrical Engineering and Informatics, Vocational College, Universitas Gadjah Mada, Yogyakarta 55281, Indonesia; 3 Sanritsu Machine Industry Co., Ltd., Chiba 263-0002, Japan; akira@sanritsu-machine.com

**Keywords:** metal particle detection, electrical impedance tomography, generative adversarial network

## Abstract

The minor copper (Cu) particles among major aluminum (Al) particles have been detected by means of an integration of a generative adversarial network and electrical impedance tomography (GAN-EIT) for a wet-type gravity vibration separator (WGS). This study solves the problem of blurred EIT reconstructed images by proposing a GAN-EIT integration system for Cu detection in WGS. GAN-EIT produces two types of images of various Cu positions among major Al particles, which are (1) the photo-based GAN-EIT images, where blurred EIT reconstructed images are enhanced by GAN based on a full set of photo images, and (2) the simulation-based GAN-EIT images. The proposed metal particle detection by GAN-EIT is applied in experiments under static conditions to investigate the performance of the metal detection method under single-layer conditions with the variation of the position of Cu particles. As a quantitative result, the images of detected Cu by GAN-EIT ${\overset{̿}{\psi }}^{GAN}$ in different positions have higher accuracy as compared to ${\left\langle \sigma^{*}\right\rangle }^{EIT}$. In the region of interest (ROI) covered by the developed linear sensor, GAN-EIT successfully reduces the Cu detection error of conventional EIT by 40% while maintaining a minimum signal-to-noise ratio (SNR) of 60 [dB]. In conclusion, GAN-EIT is capable of improving the detailed features of the reconstructed images to visualize the detected Cu effectively.

## 1. Introduction

**Figure 1:**
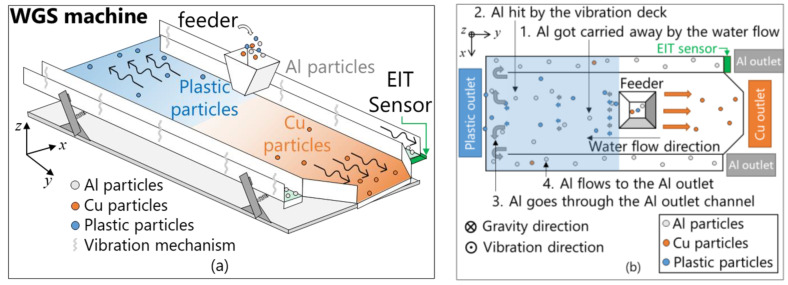
Overview of (**a**) the wet-type gravity vibration separator (WGS) and (**b**) the separation process of Al particles.

An effective recycling system is required to meet the high global demand for recycled aluminum (Al), copper (Cu), and plastic particles from electronic waste while considering economic and environmental factors [1]. In order to achieve an effective recycling system, a wet-type gravity vibration separator (WGS) is commonly provided to the recycling industry [2]. The WGS, as shown in Figure 1, has the ability to produce recycled particles with a high-purity grade from recycled materials through a separation process of an Al–Cu–plastic particle mixture [3]. The mixtures of Al, Cu, and plastic are sorted on the WGS vibration deck based on each particle’s density properties. The deck is inclined so that the Cu outlet at one end is higher than the plastic outlet at the other. The water flows from the middle of the deck toward the plastic outlet channel (see Figure 1a). Cu particles, with high-density properties, sink to the deck’s base and flow to the Cu outlet channel due to the vibration. Plastic particles, with low-density properties, float above the deck base and follow the running water to move to the plastic’s outlet channel. On the other hand, Al particles, with medium-density properties, float slightly and are still affected by water flow and vibration. The Al particles hit the back wall and go through the Al outlet channel. Finally, the Al particles flow along the channel to the Al outlet, as shown (see Figure 1b). Even though the WGS is able to produce more than 90% pure Al particles in the recycling process of Al–Cu–plastic particle mixture, the purity of separated Al particles is significantly decreased due to the minor mixed Cu particles in the Al outlet channel of the WGS. The proper parameters, such as the waste input rate, water flow rate, slope level of the deck, and vibration frequency, adjust the purity percentage, but the parameters depend on the manual operation, which requires intensive monitoring [4]. Thus, in order to raise the purity of Al particles up to 90%, it is important to detect minor Cu particles in the Al outlet channel using Cu volume fraction $\varphi^{Cu}$.

Generally, in order to detect minor Cu particles, magnetic and optical detectors have already been proposed for dry metal separators [5]. Ferrous metals can be easily separated from waste by large permanent magnets, leaving behind nonferrous metallic particles such as Cu and Al, which are mixed with nonmetallic particles such as rubber and plastic. The nonferrous metals are recovered by Eddy current separators, which use a time-varying magnetic field generated by an electromagnet to extract small particles from ore. The system recovers smaller particles more effectively without heavy rotary magnets, and the system runs in a completely dry condition to avoid wet slurry contamination [6]. However, the relative magnetic permeability of Cu, Al, and water are almost similar, which causes difficulties in detecting minor Cu particles among major Al particles in wet conditions.

On the other hand, optical detectors that use image processing to identify minor Cu particles have a significant advantage in detecting minor Cu particles [7]. The minor Cu particles in the uppermost layer are detected by image processing techniques. Still, because of the abundance of particles, metal particles flow in WGS during the separation process, producing the particles in a multi-layer configuration. The particles beneath the surface stay undetectable in this scenario. To address the image processing technique’s restriction, the impedance measurement is used to find the changes in electrical conductivity qualities between Al and Cu [8] since Al has a conductivity of $\sigma^{Cu}\;$ = 3.45 × 10^10^ mS/m while Cu has higher conductivity around $\sigma^{Cu}$ = 5.85 × 10^10^ mS/m [9] and $\sigma^{water}=$ 1 mS/m. Based on the different σ for each material, the electrical impedance measurement system has the ability to detect the $\varphi^{Cu}$ by measuring the impedance difference among Cu, Al, and water in the outlet channel of the WGS.

Under the above-mentioned situation, electrical impedance tomography (EIT), which is a non-invasive and continuous impedance measurement method, has the possibility to detect minor Cu particles. EIT has the capability to reconstruct conductivity particle distribution of a region of interest utilizing material conductivity properties [10] based on the electrical properties of the higher $\sigma^{Cu}$ than $\sigma^{Al}$ and $\sigma^{Water}$. However, EIT has a drawback to detect the minor Cu particles due to detection on the outside of the sensitivity sensing area of EIT [11], which produces a weaker signal to detect the minor Cu particles, resulting in blurry images. Hence, in order to improve the image reconstruction quality of EIT, machine learning techniques were proposed.

Machine learning enhances impedance measurement [12], especially for non-invasive imaging of the interior conductivity distribution of samples. Deep neural networks were introduced to solve the EIT problems to approximate the full inverse map to avoid the iterative solution process [13]. Another study used a machine learning adaptive electrode selection technique to build and apply a unique measurement enhancement approach to optimize electrode placements around the specimen instead of simply spacing electrodes at frequent intervals [14]. Machine learning can also be used to evaluate EIT pictures and improve the precision of EIT. A neural network-based method for the inverse problem of EIT was effective in reconstructing the conductivity distribution of a phantom object [15].

The generative adversarial network (GAN) is a generative model that creates new data similar to training data [16]. GAN has been used to recreate details of conductivity particle distribution in images reconstructed by the Landweber and Newton–Raphson techniques. The Conditional GAN (CGAN) provides detailed images, improving image quality and identification in various gas–liquid two-phase particle distributions [17]. GAN has also been used to determine phase fractions of G-L-S three-phase flows in the ECT/EMT dual-modality image fusion. The fusion model matches the phantoms under investigation, and experimental findings show appropriate particle distribution of electromagnetic characteristics [18]. In a previous study, GAN was utilized to enhance reconstructed images by leveraging the high contrast of EIT images and the sharp resolution of ultrasound images [19]. GAN has the ability to increase the quality of EIT reconstructed images by training blur images on generators and comparing them to clear images on discriminators, resulting in higher-quality reconstructed images for detection. However, unlike the referenced studies that employed close boundary sensors, our proposed methodology utilizes linear open boundary sensors.

This study solves the single-layer problem of EIT image reconstruction and proposes a deep learning approach based on GAN to estimate high-quality reconstructed EIT images from blurred images and introduce an integration system, which is GAN-EIT for $\varphi^{Cu}\;$detection in WGS. Three objectives: (1) Simulation of $\varphi^{Cu}$ in metal mixtures as the generator, (2) experimental measurement of $\varphi^{Cu}$ in the Vibration deck of WGS as the discriminator, and (3) evaluation of the significance of GAN-EIT for $\varphi^{Cu}$ measurement. The impedance measurement is conducted by injecting a constant current source and measuring the potential difference between eight channel electrodes by an adjacent measurement system. At the same time, the linear sensor is attached to the outlet of the Al channel. GAN-EIT is integrated by utilizing the reconstructed EIT images from the experiment results as input for the generator in the GAN framework, which generates enhanced images. These images are evaluated by the discriminator from the reconstructed EIT images from the simulation results to determine their similarity to the ground truth, resulting in improved image quality. The experiment is conducted in static (no vibration) conditions. Several variations of the amount of Cu and Al particles are arranged in order to mimic the actual condition. The evaluation is done by RGB comparison analysis between EIT and GAN-EIT results.

## 2. Integration of GAN and EIT to Wet-Type Gravity Vibration Separator

### 2.1. EIT Linear Sensor and Performance Test

**Figure 2:**
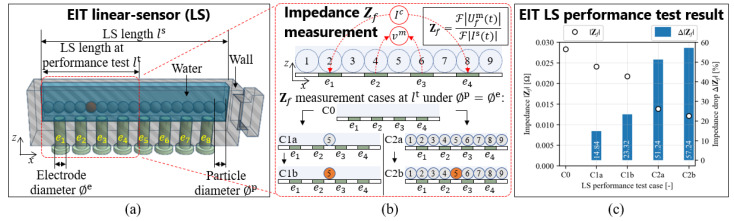
EIT linear sensor’s (**a**) design, (**b**) performance test procedure by impedance measurement, and (**c**) performance test result.

Figure 2 shows the EIT linear sensor’s (a) design, (b) performance test procedure by impedance measurement, and (c) performance test result employed in this study. As shown in Figure 2a, the EIT linear sensor features eight stainless steel electrodes with electrode conductivity $\sigma^{e}=1.47\times 10^{6}$ S/m and electrode permittivity $\varepsilon^{e}=10^{-15}$. The electrode shape is a cylinder screw with a diameter $\emptyset^{e}$ = 3 mm, which is configured the same as the round-shaped particle diameter${\;\emptyset }^{p}\;$for Al and Cu, resulting in $\emptyset^{e}=\emptyset^{Al}=\emptyset^{Cu}$. In this preliminary study, the round-shaped Al and Cu particles with a diameter of $\emptyset^{p}$ = 3 mm are utilized in order to exclude the impact of different shapes of real metal particles. Additionally, the linear sensor is enclosed; hence, no flowing particles are studied to eliminate the influence of flow variation. Thus, this research aims to investigate the characteristics of metal particles under both static conditions. The EIT linear sensor system consists of an electrode array placed in a linear arrangement, and the metal particle is put on top of the linear sensor. The linear sensor length, $l^{s}$ is adjusted to occupy as many Cu–Al particles mixture as possible in a closed-packed manner, consistent with the width of the Al outlet. As a result, the total number of particles is $N^{p}=\frac{l^{s}}{\emptyset^{p}}$. The EIT linear sensor is placed on one side of the Al outlet and attached to the WGS machine.

In order to conduct the performance test of the EIT linear sensor, an impedance measurement is conducted. As shown in Figure 2a, the area of the measurement is configured in a standard four-wire impedance measurement by employing four electrodes $e_{1},\;e_{2},{\;e}_{3,}\;$and $e_{4}$. In this case, the impedance measurement is coverage by linear sensor length at performance test$\;l_{t}$. As shown in Figure 2b, the impedance measurement is conducted by performing a fast Fourier transform (FFT) F algorithm which is expressed as [20],

(1) $$Z_{f}=\frac{F\left|U_{f}^{m}\left(t\right)\right|}{F\left|I_{f}^{s}\left(t\right)\right|}$$

where $Z_{f}\;$is the measured impedance at specific frequency f, $U_{f}^{m}\left(t\right)\;$is the measured voltage between $e_{2}\;$and $e_{3}\;$in the time domain t at f, and $I_{f}^{s}\left(t\right)\;$is the current source injection in t at f. In the performance test shown in Figure 2b, $Z_{f}$ measurement cases are divided into three categories, which are C0: water only (without particles) as a measurement’s background, C1: the C0 case with one Al (C1a) or one Cu (C1b) as an inclusion in the center of the sensor (between $e_{2}\;$and $e_{3}$), and C3: the C0 case with full-packed Al particles as an inclusion (C2a) which is modified to C2a case with a Cu particle as a replacement in the center of the sensor (C2b).

Figure 2c shows the result of the performance test conducted by performing the $Z_{f}\;$measurement in five different cases, as shown in Figure 2b. Here, we calculate the absolute $Z_{f}\;$difference between the water only $Z_{f}^{C0}$ case and after the Al/Cu inclusion in $Z_{f}^{C1a}$, $Z_{f}^{C1b}$, $Z_{f}^{C2a}$, and $Z_{f}^{C2b}$ cases as absolute impedance drop $\Delta |Z_{f}|$ by,

(2) $$\Delta |Z_{f}|=\frac{\left|Z_{f}|-|Z_{f}^{C0}\right|}{{|Z}_{f}^{C0}|}\left[100\%\right]$$

where $|Z_{f}^{C0}|\;$is the background absolute impedance, which is measured at the C0 case. As shown in Figure 2c, the EIT linear sensor has the capability to detect the single Al/Cu inclusion under the C0 case condition, as well as a single Cu inclusion under the full packed Al particles and water (C2a case). In the C1a case, the $\Delta |Z_{f}^{C1a}|\;$is dropped 14.84% because of Al inclusion; also, in the C1b case, $\Delta |Z_{f}^{C1b}|\;$is dropped 23.32% because of Cu inclusion. As well as in the C2a case, the $\Delta |Z_{f}^{C2a}|\;$dropped 51.24% because of the full-packed Al inclusion and has a countable change in the C2b case, where $\Delta |Z_{f}^{C2b}|\;$is dropped 57.24% because of single Cu inclusion among the full-packed Al and water background. In summary the $\Delta |Z_{f}^{C1a}|<\Delta |Z_{f}^{C1b}|$ and $\Delta {|Z}_{f}^{C2a}\left|<\Delta \right|Z_{f}^{C2b}|$ occurred since the $\sigma^{Al}<\sigma^{Cu}$.

### 2.2. Integration of GAN-EIT

**Figure 3:**
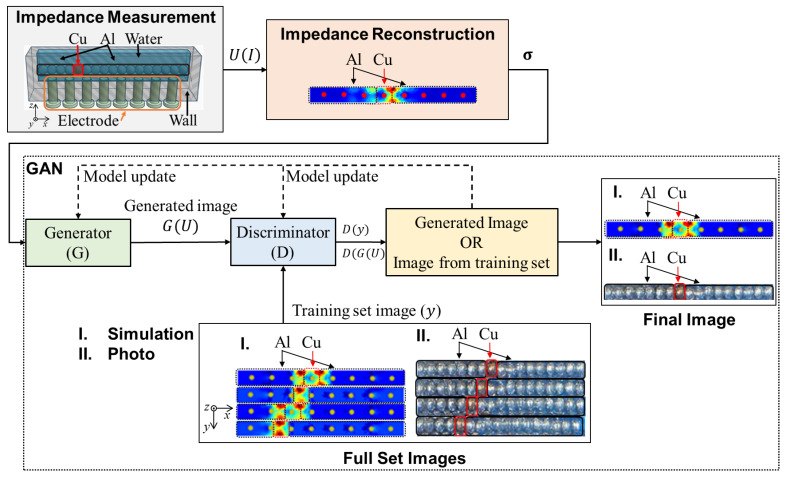
Diagram of GAN-EIT based on photo-based image and simulation-based image.

Figure 3 shows the integration of the generative adversarial network and electrical impedance tomography (GAN-EIT), which is composed of a (1) image reconstruction algorithm and a (2) GAN algorithm.

#### 2.2.1. Image Reconstruction Algorithm

The conductivity distribution σ of metal particle in sensor domain Ω for image reconstruction is expressed as

(3) $$\sigma ={\left[\sigma_{1}\left(r_{1}\right),\;\ldots ,\sigma_{n}\left(r_{n}\right),\ldots ,\;\sigma_{N}\left(r_{N}\right)\right]}^{T}∊R^{N}$$

where $r_{n}=\left(x_{n},y_{n}\right)∊R$ is the row vector at the n-th mesh point $\left(1\leq n\leq N\right)$. The value of σ is obtained using standard Jacobian matrix J, which is composed of all combinations of the current injection *I* and measured impedance Z. J is defined as $J=\left[J_{m}^{n},J_{m}^{n},\ldots ,J_{m}^{n},\ldots J_{M}^{N}\right]R^{M\times N}$, where M is the total number of measurements, and N is the total number of spatial resolutions meshes. The fundamental equation for standard Jacobian matrix element $J_{n}^{m}$ calculation of m-th pattern of measured impedance at n-th element of mesh is [21],

(4) $$J_{n}^{m}=-\int_{A}^{\;}\nabla U\left(I^{i,i+1}\right)\cdot \nabla U\left(I^{j,j+1}\right)dA\;$$

where $\gamma \left(I^{i,i+1}\right)$ is the potential field due to current I injected between the *i*-th electrode $e_{i}$ and its adjacent electrode $e_{j+1}$, where i=1, 2, …, E, $U\left(I^{j,j+1}\right)$ is the potential field due to assumed current in the case of impedance measured between remaining electrodes, which is the *j*-th electrode $e_{j}$ and its adjacent electrode $e_{j+1}$, where j=i+2,i+3,…,i+6, A is area of electric field in the sensor. For this study, the Gauss–Newton image reconstruction algorithm is used as follows [22],

(5) $$\sigma^{k+1}=\sigma^{k}+{\left(J^{T}J+\lambda R\right)}^{-1}J^{T}\Delta Z$$

where **R** is a regularization matrix; λ is a relaxation factor which is determined by the L-curve method [23], and $\Delta Z={\left[\Delta Z_{1},\ldots ,\;\Delta Z_{m},\;\ldots ,\;\Delta Z_{M}\right]}^{T}∊R^{M}$, is the normalized measured impedance under boundary shape ∂Ω which is expressed as

(6) $$\Delta Z_{m}=Z_{m}^{Al,Cu}-Z_{m}^{Al}$$

where $Z_{m}^{Al,Cu}$ is inclusion impedance based on Al–Cu mixtures, and $Z_{m}^{Al}$ represents the initial measured impedance of Al only.

The GAN algorithm models only consist of a generator and a discriminator model. The generative model is produced based on an existing dataset of EIT images and evaluated by feedback from the discriminator. The discriminative model learns the probability particle distribution based on the real image and determines whether the generated images are real ones. The data from the generator and discriminator reach a convergence state by playing a zero-sum game.

#### 2.2.2. GAN Algorithm

The GAN algorithm used in this study is based on image-to-image mappings [24]. The generator *G* maximizes the likelihood *L,* which is the function of the voltage U, and the conductivity σ, according to [25],

(7) $$L\left(G,U,\sigma \right)=\prod_{i=1}^{M}P_{G\left(V\right)}\left(\sigma^{\left(i\right)};\theta_{G}\right)$$

where P is the discrete distribution function, and the goal is to find the suitable generator parameter $\Theta_{G}^{*}$,

(8) $$\Theta_{G}^{*}=\underset{\theta_{G}}{\mathrm{argmax}}E_{\sigma }\left[\log P_{G\left(U\right)}\left(\sigma^{\left(i\right)};\theta_{G}\right)\right]$$

(9) $$\Theta_{G}^{*}=\underset{\theta_{G}}{\mathrm{argmax}}\int_{\sigma }^{\;}p_{\sigma }\left(x\right)\log p_{G\left(U\right)}(x;\theta_{G})dx-\int_{\sigma }^{\;}p_{\sigma }\left(x\right)\log p_{\sigma }(x)dx$$

where E is the mathematical expectation and p is the continuous distribution function. The term $\int_{\sigma }^{\;}p_{\sigma }\left(x\right)\log p_{\sigma }(x)dx$ removed from the previous formula, which is independent of $\theta_{G}$, must have no effect on the maximum point solution. The goal is to build the following Kullback–Leiber divergence, which is a statistical measure of probability distribution similarity.

(10) $$\Theta_{G}^{*}=\underset{\theta_{G}}{\mathrm{argmax}}\int_{\sigma }^{\;}p_{\sigma }\left(x\right)\log\frac{p(x;\theta_{G})}{p_{\sigma }(x)}dx$$

(11) $$\Theta_{G}^{*}=\underset{\theta_{G}}{\mathrm{argmin}}KL(P_{\sigma }\left(x\right)\left\|P_{G\left(U\right)}\left(x;\theta_{G}\right)\right.$$

Because the K–L divergence minimization problem cannot be solved directly with maximum likelihood, the discriminator loss is defined as follows in order to optimize the generator parameters, i.e., to calculate instead of using maximum likelihood estimation:

(12) $$Value\left(D,G\right)=\int_{\sigma }^{}p_{\sigma }\left(x\right)\log D(x)dx+\int_{V}^{}p_{U}\left(x\right)\log\left(1-D(G\left(x\right))\right)dx$$

According to the Radon–Nikodym theorem:

(13) $$Value\left(D,G\right)=\int_{\sigma }^{}\left(p_{\sigma }\left(x\right)\log D\left(x\right)+p_{G}\left(x\right)\log\left(1-D\left(x\right)\right)\right)dx$$

where $p_{\sigma }\left(x\right)$ and $p_{G}\left(x\right)$ are not affected by the discriminator $D\left(x\right)$. Let the integrand of the previous formula take the derivative of $D\left(x\right)$ and set it to 0:

(14) $$\frac{p_{\sigma }\left(x\right)}{D(x)}+\frac{p_{G}(x)}{D\left(x\right)-1}=0⟹D\left(x\right)=\frac{p_{\sigma }\left(x\right)}{p_{\sigma }\left(x\right)+p_{G}\left(x\right)}$$

It can be demonstrated that if and only if $p_{\sigma }\left(x\right)=p_{G}\left(x\right),\;D\left(x\right)=0.5$, the reconstructed conductivity calculated by the generator is exactly consistent with the real conductivity, and the discriminator cannot tell whether the image is from the generator or real samples. In this article, the GAN generator and discriminator are both back-propagation neural networks that adapt to voltage and conductivity vectors. This GAN model’s goal function is:

(15) $$\underset{\theta_{G}}{\min}\underset{\theta_{D}}{\max}\;Value\left(D,\;G\right)=E_{\sigma }\left[\log D\left(\sigma \right)\right]+E_{V}\left[\log(1-D\left(G(U))\right)\right]$$

where D(x) is the discriminator and G(z) is the generator. While Px is the real data particle distribution, Pz is the particle distribution of the generated data. The discriminator D(x) is trained to maximize its ability to decide whether the generator output is real or not while the generator G(z) is trained to minimize the output. When the real particle distribution is equivalent to the generated particle distribution, the output by the discriminator is considered as the optimal result.

The ideal image is the reference image, which is obtained from image processing or simulation. The blurred images from the reconstructed image serve as the training set for the generative model. The generated image is then investigated by the discriminative model to determine whether the image is equivalent to the ground truth. When the data particle distribution of the discriminator is equivalent to the data particle distribution of the generator, the output of the discriminator is defined as the optimal result.

#### 2.2.3. Evaluation Metric

To assess the effectiveness of GAN-EIT, we measured the similarity between the GAN-EIT image and the ground truth using the Pearson correlation coefficient (PCC) [26] and structural similarity indices (SSIM) [27]. PCC is a statistical measure of the strength of the linear relationship between two variables and determines how related two variables are to each other. The Pearson correlation coefficient (PCC) ranges from −1 to 1, where −1 indicates a perfectly negative correlation, 0 indicates no correlation, and 1 indicates a perfectly positive correlation. The PCC can be expressed as,

(16) $$PCC=\frac{\sum_{i=1}^{N}\left(y_{i}-\bar{y})(y_{i}^{\prime }-\bar{y\prime }\right)\;}{\sum_{i=1}^{N}{\left(y_{i}-\bar{y}\right)}^{2}\sum_{i=1}^{N}{\left(y_{i}^{\prime }-\bar{y\prime }\right)}^{2}}$$

where $y_{i}$ is the intensity of the i-th pixel in ground truth image, $y_{i}^{\prime }$ is the intensity of the i-th pixel in GAN-EIT image, $\bar{y}$ is the mean of $y_{i}$, and $\bar{y\prime }$ is the mean of $y_{i}^{\prime }$.

The SSIM indicates the nonlinear change between GAN-EIT images and the ground truth images [28], which is defined as

(17) $$SSIM\left(y,y^{\prime }\right)=\frac{\left(2\cdot \bar{y}\bar{y\prime }+c_{1}\right)\cdot (2\cdot \mu_{yy^{\prime }}+c2)\;}{\left({\bar{y}}^{2}+{\bar{y^{\prime }}}^{2}+c_{1}\right)\cdot \left(\mu_{y}^{2}+\mu_{y^{\prime }}^{2}+c_{2}\right)}$$

where $\mu_{y}$ and $\mu_{y^{\prime }}$ denote the standard deviation of y and y′, and $\mu_{yy^{\prime }}$ is the covariance of both images. The addition of variables $c_{1}$ and $c_{2}$ stabilizes the division with a weak denominator. Higher SSIM values indicate better image synthesis [29].

## 3. Simulation

### 3.1. Preparation of Full Set Photo Images

**Figure 4:**
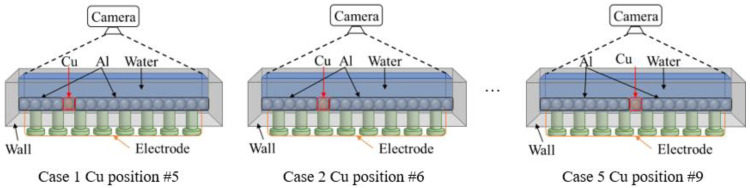
Cases for full set photo images.

Figure 4 shows cases for full sets of photo images. The image was captured by putting a camera on top of the linear sensor to get the top view of metal particles inside the linear sensor. Each image is obtained with different positions of Cu particles. Cu position from 5 to 9 is investigated as the preliminary study.

### 3.2. Preparation of Full Set Simulation Images

#### 3.2.1. Simulation Method

Numerical simulation studies are employed by a finite element method (FEM) software based on COMSOL Multiphysics v5.3a with AC/DC module at stationary study in order to generate input for training data set [30]. The simulation of electric potential $\phi \left(r\right)$ inside a subdomain Ω is produced by placing a current across the surface in boundary ∂Ω on each electrode transmitter with the injected current i [31].

(18) $$\nabla \cdot \left(\sigma^{*}\left(r\right)\right)\nabla \phi \left(r\right)=0,\;r\in \Omega$$

(19) $$\phi \left(r\right)+Z_{c}\sigma^{*}\left(r\right)\frac{\partial \phi \left(r\right)}{\partial n}=U_{l},r\in e_{l},l=\left\{1,\;\ldots ,\;L\right\}$$

(20) $$\int_{\partial \Omega^{e_{l}}}^{\;}\sigma^{*}\left(r\right)\frac{\partial \phi \left(r\right)}{\partial n}dS=I,r\in \partial \Omega^{e_{l}}$$

(21) $$\sigma^{*}\left(r\right)\frac{\partial \phi \left(r\right)}{\partial n}=0,r\in \partial \Omega \backslash \overset{L}{\underset{l=1}{⋃}}e_{l}$$

where, $\sigma^{*}∶=\sigma +2\pi f\varepsilon \;\in C[Sm^{-1}]$ is the non-homogenous admittivity property of metal, σ and ε are the conductivity and absolute permittivity [Fm^−1^] respectively in Ω at the frequency f, $\phi \left(r\right)\;\in C[V]$ is the electric potential particle distribution, and $r∶=\left(x,y,z\right)$ is the coordinate system in subdomain Ω.

#### 3.2.2. Simulation Condition

The simulation focuses on the displacement of particles in a three-dimensional (3D) model under static conditions. Since the measurement technique is based on four-wire impedance measurement, the 3D model is constructed by considering the configuration of each of the four electrodes and the particles in the measurement domain Ω. The simulation creates reference images of Al–Cu mixtures by varying the Cu positions in the static condition. Here, the linear sensor consisted of eight electrodes with a size of 51 × 3 × 10 mm. The particle diameter $\emptyset_{p}$ remains constant and configured the same with the electrode diameter $\emptyset_{e}$ = 3 mm. In order to assess the measurement under submerged particle condition, the water layer height $h_{w}$ is adjusted to 0.75$\emptyset_{p}$. The conductivity value of Al particles is set at $\sigma^{Al}\;$3.45 × 10^10^ mS/m, Cu particles is at $\sigma^{Cu}$ = 5.99 × 10^10^ mS/m and water are at $\sigma^{w}$ = 1 mS/m. In addition, on the boundary condition setting, a constant current injection on injecting electrode $e_{1\;}$is I = 1 [mA] with frequency f = 2 [kHz]. Meanwhile, the ground electrode is set to$\;e_{4}$ and floating potential electrodes are set to $e_{2}\;$and $e_{3},$ respectively.

#### 3.2.3. Simulation Result

**Figure 5:**
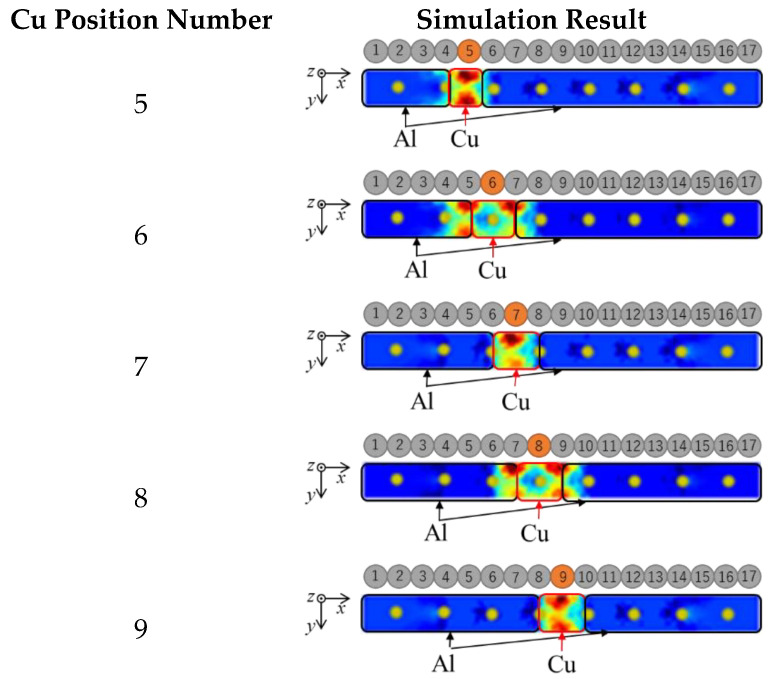
Simulation results on several Cu positions of all 17 positions.

Figure 5 shows the image reconstruction based on simulation results in static conditions. Electrical impedance tomography (EIT) has been used effectively to generate images based on simulation results. The reconstructed EIT images successfully distinguished Cu (red) in various positions according to different Cu position numbers as in the illustration, demonstrating EIT’s accuracy in capturing and representing changes in electrical impedance within Al–Cu mixtures. These reconstructed images are further employed as inputs for the Generative Adversarial Network (GAN) model, improving the quality of reconstructed images.

## 4. Experiment

### 4.1. Experimental Setup

**Figure 6:**
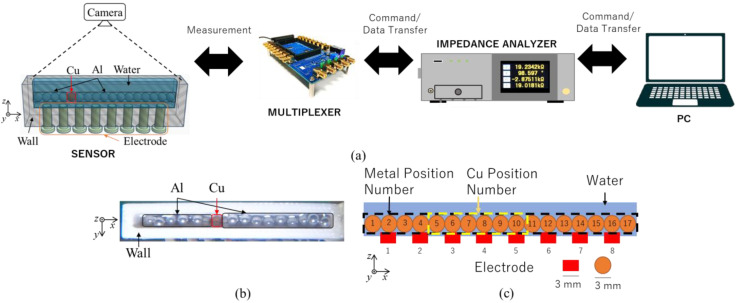
(**a**) Experimental setup, (**b**) Top-view real image of Al–Cu mixtures where Cu is in position 7, (**c**) Diagram of linear sensor setup by eight-electrodes and Cu position variation from number 1 to 17.

Figure 6a shows the experimental setup of the EIT system, which is composed of a linear sensor, an eight-channel multiplexer, an impedance analyzer (IM 3570, Hioki E.E. Corporation, Tokyo, Japan), and a PC. The linear sensor was manufactured from polylactic acid (PLA) with the dimension of the container 51 × 3 × 10 mm and consisted of eight electrodes. Stainless steel screws with 3 mm in diameter were used as the electrodes linear sensor with the gap between each of 3 mm. The shape and arrangement of the linear sensor were built as a preliminary investigation of measuring the conductivity of Cu. The impedance analyzer measures the impedance from different voltages from the linear sensor. As a device for switching between electrodes, the multiplexer switches the acting electrodes based on the neighboring measurement pattern. The PC in this system manages all measuring methods and gathers data for subsequent analysis. The conductivity distribution between the Cu and Al particle mixes is then visualized by an image reconstruction approach. As a result, the Cu particles are distinguished from the Cu–Al particle mixtures. The command and image reconstruction are done from the PC. In the post-processing, the GAN algorithm is applied to improve the reconstructed image quality.

### 4.2. Experimental Conditions

The current applied in the measurement of AC current is I=1mA with a fixed frequency of 2 kHz. In the static condition experiment, vibration from the metal separator was neglected. A static condition experiment was done in order to understand two things: basic physics of measurement and linear sensor evaluation. The ideal particle is preferred to the real particle in preliminary experiments. The ideal particle is Al and Cu balls with a diameter of 3 mm, while real particles have a tiny size in millimeter order and random shape. This study investigated the metal behavior under a 1-layer condition. Figure 6b,c show the variation of position and number of Cu particles to determine the sensing area of the linear sensor. Cu position from 5 to 9 is investigated as the preliminary study.

### 4.3. Experimental Method

**Figure 7:**
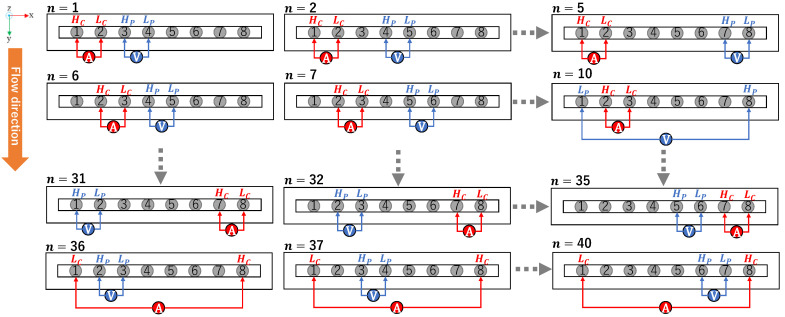
Adjacent measurement pattern where V is the voltage measurement and A is the current injection.

Figure 7 shows the adjacent measurement pattern where each electrode in the linear sensor was taking turns to act as the $H_{c},\;{\;L}_{c},\;{\;H}_{p},\;$and $L_{p}$ electrode. An adjacent injection-measurement pattern is used in this study. Here, for each measurement number (n), one electrode acts as the current injector or high current ($H_{c}$), the injected current then flows through the ground or low current ($L_{c}$) electrode, and the voltage generated by the flowing current is then measured by pair of electrodes acting as high potential ($H_{p}$) and low potential ($L_{p}$).

### 4.4. Experimental Results

**Figure 8:**
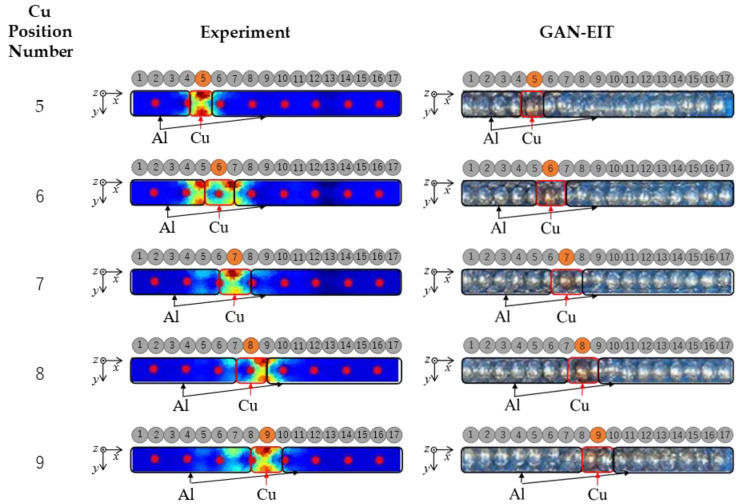
Comparison results of reconstructed images in static conditions on several Cu positions of all 17 positions.

The electrical impedance tomography (EIT) technique has been successfully utilized to reconstruct images based on experimental results. However, as shown in Figure 8, the reconstructed EIT images were found to be blurry, making it difficult to distinguish between the different Cu positions within Al–Cu mixtures. These blurry images were further used as inputs for the Generative Adversarial Network (GAN) model to enhance their quality and improve their visual clarity. The results obtained from the GAN-EIT integration demonstrated that the GAN model was successful in enhancing the images obtained from EIT, resulting in images that were more feasible to distinguish between the different Cu positions within Al–Cu mixtures. These findings highlight the potential of integrating EIT with GAN for enhancing the image quality and improving the interpretability of the reconstructed images in experimental settings. The results of this study underscore the promising potential of the GAN-EIT integration and its relevance for further research and practical applications.

## 5. Discussion

### 5.1. EIT Analysis

**Figure 9:**
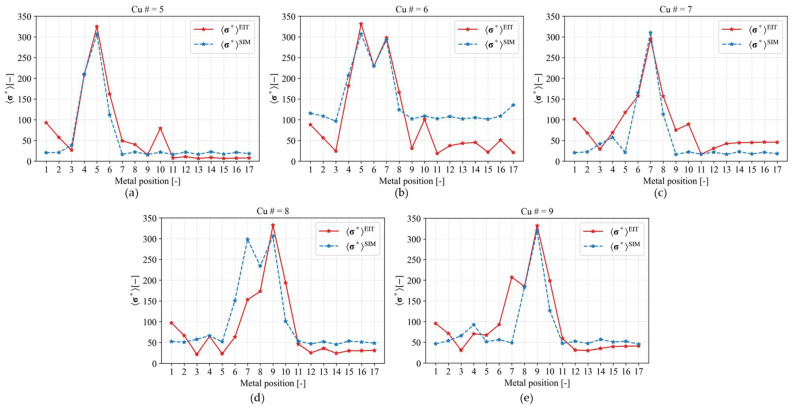
Comparison of normalized spatial mean conductivity distribution under simulations and experiments for Cu position (**a**) 5, (**b**) 6, (**c**) 7, (**d**) 8, and (**e**) 9.

Figure 9 shows the comparison of spatial mean conductivity distribution $\left\langle \sigma^{*}\right\rangle$ under simulation ${\left\langle \sigma^{*}\right\rangle }^{SIM}\;$and an experiment using EIT ${\left\langle \sigma^{*}\right\rangle }^{EIT}$, which represent the relationship between $\left\langle \sigma^{*}\right\rangle$ and metal position in the case of different Cu position (5–9). The $\left\langle \sigma^{*}\right\rangle$ represent spatial mean conductivity distribution in the sensor domain. The relationship between $\left\langle \sigma^{*}\right\rangle$ and metal position is successfully recognized for Cu position in 5, 7, and 9 for both simulation and experiment, which are shown by the highest $\left\langle \sigma^{*}\right\rangle$ on each plot. However, in the case of the Cu position at 6, both simulation and experiment results fail to recognize the Cu position correctly as in fact, the highest $\left\langle \sigma^{*}\right\rangle$ is shown in metal position 5 and 7, meanwhile, for the Cu position at 8, the ${\left\langle \sigma^{*}\right\rangle }^{EIT}$ shows metal position 9 as the highest $\left\langle \sigma^{*}\right\rangle$, while the ${\left\langle \sigma^{*}\right\rangle }^{SIM}$ shows metal position 7 and 9 as the highest $\left\langle \sigma^{*}\right\rangle$. One of the reasons is that the even Cu position is located on top of the electrode which generates image artifacts. On the other hand, the odd Cu position is located between electrodes, which generates correct images. Overall, EIT successfully reconstructed images of several Cu positions but remains unreliable as a single modality for a metal position detection method.

### 5.2. GAN-EIT Image Evaluation

**Figure 10:**
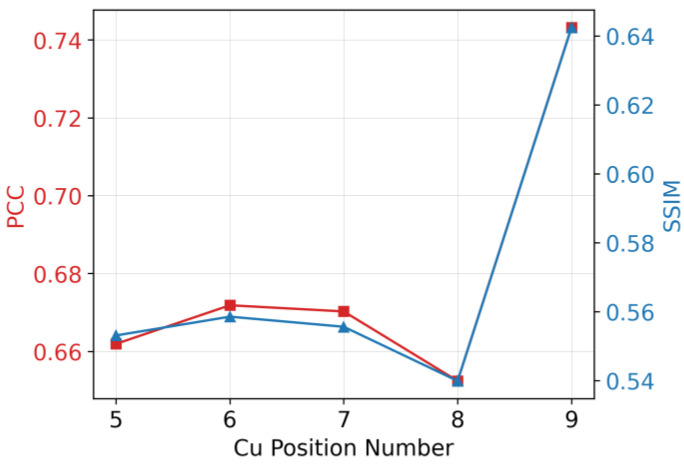
Comparison of PCC (red) and SSIM (blue) results.

The evaluation of the GAN-EIT image is shown in Figure 10. In summary, based on the PCC values, Cu positions number 5–8 demonstrate moderate positive linear correlations with their respective counterparts. Conversely, Cu position number 9 exhibits a relatively strong positive linear correlation. Meanwhile, based on the SSIM values, all image pairs represented by these variables display moderate levels of structural and perceptual similarity, with Cu position number 9 showcasing the highest level of similarity.

### 5.3. Cu Detection Based on GAN-EIT Images Using RGB Analysis

**Figure 11:**
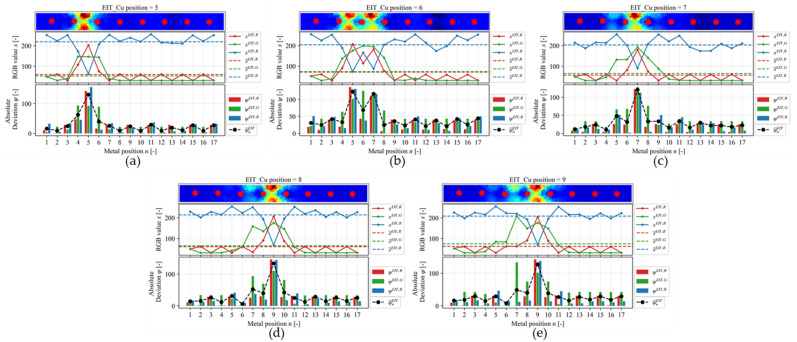
The RGB analysis on EIT image reconstruction results for Cu position (**a**) 5, (**b**) 6, (**c**) 7, (**d**) 8, and (**e**) 9.

**Figure 12:**
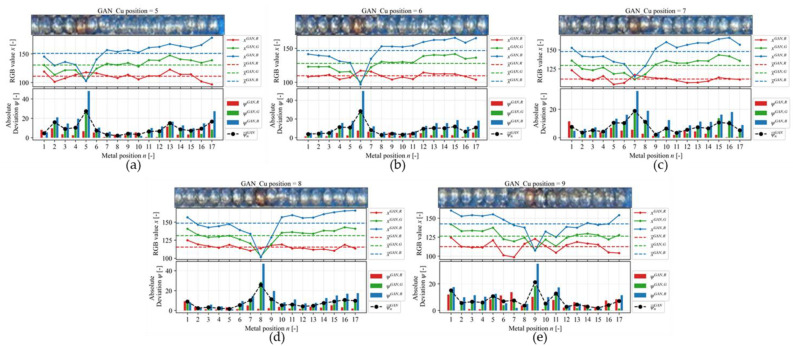
The RGB analysis on GAN-EIT image reconstruction results for Cu position (**a**) 5, (**b**) 6, (**c**) 7, (**d**) 8, and (**e**) 9.

The evaluation of performance between GAN-EIT and original EIT is performed by RGB value analysis. Each image is represented by red, green, and blue (RGB) color values.

(22) $$\psi_{n}^{\alpha ,\beta }=\left|x_{n}^{\alpha ,\beta }-{\bar{x}}^{\alpha ,\beta }\right|$$

(23) $${\bar{\psi }}_{n}^{\alpha }=\frac{1}{3}\psi_{n}^{\alpha ,R}+\psi_{n}^{\alpha ,G}+\psi_{n}^{\alpha ,B}$$

where x is the color intensity value of every pixel in one of RGB channels, $\bar{x}$ is the average of x, ψ is the absolute deviation, $\bar{\psi }$ is the mean absolute deviation, $\alpha =\left\{EIT,GAN\right\}$ is the method used to generate the image, $\beta =\left\{R,G,B\right\}$ is the color intensity of every pixel in the three different red (R), green (G), and blue (B) channels, $n=\left\{1,\ldots ,17\right\}$ is the number of position of the metal in the linear sensor. Figure 11 shows the RGB analysis on the EIT image reconstruction result with Cu in different positions, while Figure 12 shows the RGB analysis of the GAN-EIT image reconstruction result with Cu in different positions. The relationship between ψ and metal position is successfully recognized for Cu position in 5, 7, and 9 for EIT, which is shown by the highest ${\bar{\psi }}^{EIT}$ on each plot. As already discussed in the previous analysis, in the case of Cu positions 6 and 8, ${\bar{\psi }}^{EIT}$ shows low accuracy as it detects the adjacent positions as well. The low accuracy of linear sensor EIT is due to several drawbacks, such as contact impedance [32], stray capacitance [33], and measurement noise [34]. On the other hand, the GAN-EIT detects all Cu positions, which are shown by the highest ${\bar{\psi }}^{GAN}$ on each plot. Overall, GAN-EIT successfully reconstructed images of all Cu positions accurately. In comparison, the RGB analysis on EIT showed the Cu position with low accuracy, whereas the GAN-EIT image showed the Cu position with high accuracy.

**Figure 13:**
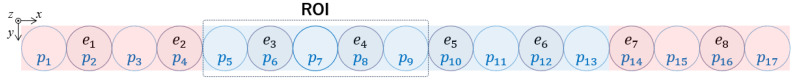
The blue shading shows the location of the copper particles that often passed before exiting the Al outlet channel. The region of interest (ROI) shows the optimum detected area of the linear sensor.

**Figure 14:**
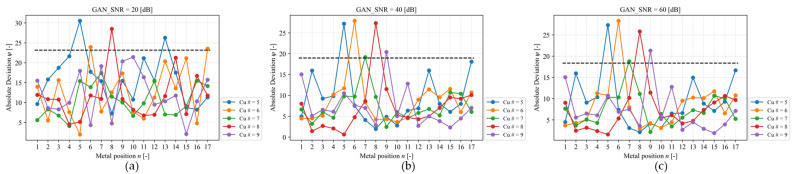
Cu detection based on ψ for Cu position = 5 (blue); 6 (orange); 7 (green); 8 (red); and 9 (purple) with different noise signals, (**a**) 20 [dB] (the worst condition), (**b**) 40 [dB], and (**c**) 60 [dB]. The black line shows the 95% quartile threshold.

The limitation of EIT using the adjacent pattern is that in the case electrode 1 acts as the high-current electrode and electrode 2 acts as the low-current electrode, four metal particles on top of them are neglected because the voltage measurement, which detects the metal particle, is done by the other electrodes. Along with the measurement, the optimum metal particles that are able to be detected are in positions 5–9. In the case of the electrode arrangement being flipped, the metal particles in positions 10–13 are detectable. Copper particles mostly pass through the center of the mouth’s channel (area shown in Figure 13). Figure 14 shows Cu detection based on absolute deviation for different Cu positions with different noise signal conditions. In the worst case (Figure 14a), a noise signal with 20 [dB] was not significant to distinguish different Cu positions. At SNR = 60 [dB] (Figure 14c) shows that Cu positions 5–9 are distinguishable above the 95% quartile threshold. The linear sensor designed for the area of interest (ROI) effectively reduces the detection error of copper (Cu) in conventional electrical impedance tomography (EIT) by 40%. This improvement is achieved while ensuring a minimum signal-to-noise ratio (SNR) of 60 [dB] in the presence of additive white Gaussian noise (AWGN) [8].

In summary, GAN-EIT is a promising approach for improving the quality of EIT reconstructed images. The use of GANs enables the development of high-quality images which are more similar to ground truth images. The effectiveness of the EIT-GAN technique, however, is dependent on several aspects, including the quality and amount of EIT data, the reconstruction algorithm chosen, the GAN architecture and hyperparameters, and the assessment metrics and criteria. Further research is needed to explore the full potential and limitations of this approach, as well as to develop novel methods and applications that benefit from the integration of EIT and GAN.

This study has determined that the proposed GAN-EIT has a low error percentage in detecting Cu particles among major Al particles. Therefore, it is important to consider the robustness of our method when applying it to the metal separation process using a wet gravity separator, as this process involves extreme conditions caused by the machine’s vibration and mixed particle flow. Hence, we plan to investigate our findings under these conditions in future research.

## 6. Conclusions

In this study, we have investigated the integration of electrical impedance tomography (EIT) with Generative Adversarial Networks (deGAN) for image reconstruction and enhancement in order to distinguish minor Cu particles from major Al particles for a wet-type gravity vibration separator. The results obtained from our experiments have provided valuable insights into the feasibility of this integration in improving the quality and interpretability of EIT reconstructed images. Our findings indicate EIT has been successfully utilized to generate images based on simulation/experimental results, but the reconstructed images were often blurry and lacked clarity, making it difficult to distinguish between the different Cu positions within Al-Cu mixtures. However, through the integration of GAN-EIT, we were able to enhance the images obtained from EIT, resulting in images that exhibited improved visual fidelity and clarity.

The GAN-EIT model effectively improved the quality of the reconstructed images, making them more feasible to interpret and analyze the different Cu positions within Al–Cu mixtures. The conclusions are summarized as follows:

1. GAN-EIT reduces Cu detection error of conventional EIT by 40%.
2. The proposed method is reasonably robust to prevent intervention from the noise signal condition of a device. An impedance measurement with a minimum SNR = 60 [dB] is recommended.
3. GAN-EIT is capable of improving the detailed features of the EIT images to detect Cu effectively.

## Data Availability

Not applicable.
